# Doctors of Medical Science

**Published:** 1883-02

**Authors:** 


### Doctors of Medical Science.

The French Minister of Public Instruction has recently issued a circular to the doctors of the medical faculties and schools, requesting them to seek the advice of their various councils as to whether it would be desirable to create a new degree superior to that of Doctor of Medicine, to be termed Docteur des Sciences Medicales. While leaving the discussion on the subject free and open to the profession and academic councils, and desiring their opinion on the matter, the Minister observes that there are three points to be especially considered: 1. The utility of having above the doctorate of medicine, which is especially a professional degree, a superior degree indicating more complete and more scientific acquisitions, and more personal and more original studies. 2. What exigencies should be imposed on the candidates for the new doctorate beyond those already required for the doctorate of medicine? 3. In what way are the tests of the qualifications of the candidates to be settled?

